# Assessing Orofacial Pain Behaviors in Animal Models: A Review

**DOI:** 10.3390/brainsci13030390

**Published:** 2023-02-24

**Authors:** Sufang Liu, Joshua Crawford, Feng Tao

**Affiliations:** Department of Biomedical Sciences, Texas A&M University College of Dentistry, Dallas, TX 75246, USA

**Keywords:** orofacial pain, behavioral testing, rodents, animal models, evoked pain, spontaneous pain, ongoing pain

## Abstract

Orofacial pain refers to pain occurring in the head and face, which is highly prevalent and represents a challenge to clinicians, but its underlying mechanisms are not fully understood, and more studies using animal models are urgently needed. Currently, there are different assessment methods for analyzing orofacial pain behaviors in animal models. In order to minimize the number of animals used and maximize animal welfare, selecting appropriate assessment methods can avoid repeated testing and improve the reliability and accuracy of research data. Here, we summarize different methods for assessing spontaneous pain, evoked pain, and relevant accompanying dysfunction, and discuss their advantages and disadvantages. While the behaviors of orofacial pain in rodents are not exactly equivalent to the symptoms displayed in patients with orofacial pain, animal models and pain behavioral assessments have advanced our understanding of the pathogenesis of such pain.

## 1. Introduction

Orofacial pain includes pain in the face and head. Headache mainly occurs in the orbit and/or above the nape crest, while facial pain refers to pain below the orbital line, in front of the auricle, and above the neck [1]. The incidence of orofacial pain in the United States is about 21.7%, and its treatment is still not satisfactory [2]. As an unpleasant sensory and emotional experience, pain is usually divided into two categories: “spontaneous pain” (stimulus-independent) and “induced or evoked pain” (stimulus-dependent) [3,4]. The latter includes allodynia and hyperalgesia. To understand the underlying mechanisms for different types of pain, we need to measure pain behaviors in animal models using specific behavioral testing methods. Currently, rodents are the most commonly used for preparing orofacial pain animal models. In this review, we will summarize various behavioral testing methods for measuring orofacial pain in rodent models and discuss their advantages and disadvantages. According to the published information described here, pain researchers may choose appropriate methods to test pain behaviors in their future studies. 

## 2. Behavioral Assessment of Spontaneous Pain

Spontaneous pain is indistinguishable from ongoing pain in the inflammatory state. Strictly speaking, spontaneous pain refers to the spontaneous firing of neurons in the somatosensory system. As pain is a subjective experience, specific methods need to be used to determine the extent of spontaneous pain in rodents. Commonly used methods include the grimace scale, grooming, and conditioned or real-time place preferences. These methods have their advantages and disadvantages, and different methods can be chosen according to study objectives and animal models used.

### 2.1. Grimace Scale

The grimace scale was first created to detect pain in mice [5]. In short, the facial expressions of mice were observed following an intraperitoneal injection of 0.9% acetic acid to stimulate abdominal contraction and induce pain. Mice were individually placed in a plexiglass chamber and images were captured with a digital camera every three minutes for 30 min before and after the painful stimulus. Mouse facial expression includes five characteristics: orbital tightening, nose bulge, check bulge, ear position, and whisker change, which are given a 0 (not present), 1 (moderately visible), or 2 (severe) for each facial expression [5]. This research group indicates that the mouse grimace scale (MGS) is a sensitive method for testing inflammatory pain but may not be suitable for measuring hind paw damage-caused pain [5]. 

One year after the creation of MGS, the rat grimace scale (RGS) was developed [6]. The researchers induced pain by injecting 150 µL of 50% CFA into the plantar surface of the rat hind paw and then recorded the RGS at different time points for 30 min. Rodent Face Finder^®^ (RFF) software was used to extract image frames every 3 min for data analysis. The observation indicators are slightly different from MGS. The facial expression assessed in the RGS includes four characteristics: orbital tightening, nose/cheek flattening, ear changes, and whisker changes. The scoring standard is the same as that in the MGS, including 0, 1, and 2 three-level scorings [6]. 

*Use of the grimace scale in testing temporomandibular disorders (TMDs):* TMDs are disorders of the jaw muscles, temporomandibular joint (TMJ), and related nerves [7], which are often accompanied by TMJ pain and headache, as well as dysfunction of the TMJ and its associated tissue structure. While animal models of TMD can be prepared using chemical, mechanical, or surgical approaches [8], the use of MGS or RGS has been best utilized in measuring spontaneous pain in chemically induced TMD models.

In our previous study, MGS was used to evaluate the degree of TMJ pain induced by intra-TMJ injection of complete Freund’s adjuvant (CFA) in mice. By recording a 30-min video with a high-resolution digital camera before and after CFA or saline injection into the TMJ, we evaluated the orbital tightening, nose bulge, and ear position changes. We observed that the MGS score in the CFA-treated mice was significantly higher than that in the control group. Interestingly, the change in orbital tightening is the most durable and obvious indicator for measuring such inflammatory TMJ pain [9]. In another TMJ pain mouse model with masseter muscle tendon ligation, the MGS score was significantly increased compared to the sham group [10]. In addition, CFA injection into the masseter muscle also increased the MGS score remarkably compared to the saline control group [11,12]. To date, the grimace scale is much more often used in mouse models. In rats, Asgar et al. reported that 50 µL of 50% CFA injection into the masseter muscle produced a significant increase in the RGS score [13].

*Use of the grimace scale in testing neuropathic pain caused by chronic constriction injury of the infraorbital nerve (CCI-ION):* Previous studies have shown that MGS scores have significant changes in trigeminal neuropathic pain models in both mice and rats [14,15], while other studies have used the grimace scale method to evaluate cervical neuropathic pain in rodents [16,17,18]. 

*The advantages and disadvantages of the grimace scale method:* The advantages include: (1) It can directly indicate spontaneous pain; and (2) the results show a significant difference between pain and non-pain control groups in inflammatory TMJ pain models; (3) it is applicable to both rats and mice; and (4) the method is easy to use. The disadvantages include: (1) It is difficult to judge the facial expressions in black or gray mice and rats; (2) the scores are based on subjective observation.

The predictive validity of using the grimace scale to measure pain behaviors is moderate, and grimace scoring can be used to indicate the affective component of pain. However, the grimace scale is a reliable method for detecting the onset of pain, but additional behavioral methods may be needed to test the maintenance of pain [16].

### 2.2. Conditioned Place Preference (CPP) and Real-Time Place Preference (RTPP)

CPP was first developed to detect the preference or aversion to drugs in animals [19,20]. It was not until 1994 that CPP was used to test the effects of analgesic drugs [21]. Since then, many studies have begun to use it as a method to measure spontaneous pain [22,23,24,25]. Typically, the CPP box contains three different chambers, two of which with differently patterned walls or floors are used for different stimuli or drug/vehicle injections at each end, and a buffer room in the middle is used for transition [21,24,26]. The CPP method has been widely used to test orofacial ongoing pain in established animal models of inflamed masseter myalgia and trigeminal neuropathic pain [11,24,27,28,29,30], and among them, using CPP to measure ongoing pain behaviors in trigeminal neuropathic pain models is more popular. In our study [24], we performed a CPP test in a CCI-ION-induced trigeminal neuropathic pain mouse model. The CPP test includes three phases: preconditioning phase (days 1–3), conditioning phase (days 4–6), and testing phase (day 7) (Table 1). 

In the preconditioning phase, mice were allowed to move freely for 15 min per day in the three chambers, and the time spent in each chamber was recorded, and the result on the last day of the preconditioning phase was used as a baseline. Next, the mice were paired with optogenetic stimulation for 30 min per day in the conditioning phase. We assigned one chamber where mice preferred to stay in the preconditioning phase as the pairing chamber so that we could test whether the optogenetic stimulation would change the place preference of mice in the CPP box. After a 3-day pairing in the conditioning phase, a 15 min test was performed in the testing phase, in which mice had free access to all the chambers. An increased time spent in the pairing chamber during the 15 min test indicates that the paired optogenetic stimulation alleviates ongoing spontaneous pain in the CCI-ION-induced trigeminal neuropathic pain mouse model [24]. On the other hand, a decreased time spent in the pairing chamber during the 15 min test indicates that the paired optogenetic stimulation exacerbates the CCI-ION-induced spontaneous pain. 

To detect real-time spontaneous pain in this model, we employed RTPP in our recent study [31]. Unlike CPP, the RTPP test does not require pairing animals with stimuli. In the RTPP test, mice were measured for 15 min baseline behaviors as described in the CPP test, and then the mice were stimulated when they entered the designated chamber, while the mice were not stimulated in other chambers, including the buffer area. The total test time is 15 min. We determined the spontaneous pain in the CCI-ION mouse model by analyzing the changes in the time spent in the stimulation chamber [31].

*The advantages and disadvantages of CPP and RTPP methods:* The advantages include: (1) It can objectively detect ongoing or spontaneous orofacial pain; and (2) it is convenient to observe the influence of stimulation on such pain. The disadvantages include: (1) animal movement ability may affect the results of the two tests; and (2) drug addiction may be involved in the behavioral changes as a possible covariate.

The CPP/RTPP can indeed be used as an effective method for spontaneous pain measurement, but the limitation is that the animal may have preferences for two different chambers before receiving paired treatment, which may affect the final experimental results. In addition, some studies have shown that the use of CPP to detect human addiction could be controversial [32,33].

### 2.3. Facial Grooming

Generally, facial grooming behaviors include facial scratching, licking, and rubbing [34]. Facial grooming has been used as a spontaneous orofacial pain indicator in the following animal models: inflammatory orofacial pain caused by formalin injection into infraorbital nerve-innervated territory and CFA injection into the masseter muscle, as well as neuropathic orofacial pain caused by infraorbital nerve injury [34,35,36,37,38]. It is important to note that not all face-washing strokes are considered a feature of spontaneous pain because, under normal circumstances, animals show normal painless grooming behavior. However, compared with facial grooming caused by spontaneous pain, normal grooming usually lasts a short time and is not asymmetric [37]. 

Parameters showing the strength of facial grooming include the size of the area where the animal is scratching on the face, the duration of the scratching, and how often it occurs [35,36,37,38]. Animal scratching is scored at three levels: large (if the scratching behaviors are directed to the hairy skin area above the eyes), medium (if the scratching behaviors are directed to the hairy skin area below the eyes, extending to the area innervated by the infraorbital nerve), and small (if the scratching behaviors are restricted to the hairy skin below the vibrissae) [36]. Specifically, it is reported that in the formalin-induced orofacial pain animal model, the dose and concentration of formalin are directly proportional to the time and amplitude of animal grooming [38].

*The advantages and disadvantages of the facial grooming method:* The advantages include: (1) Only a regular camera is needed for recording animal grooming behaviors; (2) the duration, frequency, and area size of animal scratching can be measured; (3) the observation index is simple and intuitive; and (4) it can be used in both inflammatory and neuropathic orofacial pain. The disadvantages include: (1) researchers need to identify the difference between normal face washing and grooming caused by pain; (2) the scoring is subjective; and (3) the size of the scratching area can change at different time points.

Facial grooming is highly reliable in predicting postoperative craniofacial pain, independent of mouse strain, sex, and housing environment. Additionally, the grooming behavior test also shows validity for pain in an abdominal surgery model [39,40]. 

## 3. Behavioral Assessment of Evoked Pain

Evoked pain refers to hyperalgesia or allodynia caused by a specifically applied stimulus. Hyperalgesia is an increased pain response to noxious stimuli, while allodynia is a pain response to normal, innocuous stimuli [41]. Pain-induced withdrawal behavior is a reflexive reaction accompanying the pain [42]. 

### 3.1. Von Frey Test 

The most widely used method for assessing evoked pain is testing the mechanical withdrawal response with von Frey filaments, which was created by Dr. Maximilian von Frey, an Austrian-German physiologist [43]. In this method, a series of calibrated von Frey filaments with increasing diameter/force are poked onto the skin of animals until they bend slightly, and the animals’ withdrawal responses are observed. Either withdrawal threshold (smallest force to induce withdrawal) or withdrawal frequency (withdrawal times for a certain force of von Frey filament) can be measured to indicate pain severity in animal models. Thus, a decreased withdrawal threshold or increased withdrawal frequency indicates an enhanced pain behavior. The von Frey test can be performed manually or with an electronic device, which has become the most widely used approach to determine allodynia and hyperalgesia in the head and face [44]. In our previous studies, the von Frey test has been used to assess evoked pain in different inflammatory pain or neuropathic orofacial pain mouse models [9,24,31,45,46,47,48]. 

While the von Frey test has been easily utilized to measure paw withdrawal as a pain response, utilizing this test on the head and face to assess orofacial pain is difficult for the following reasons: (1) Animals can see the von Frey filament clearly during the test; (2) the filament stimulation in vibrissae easily produces a false-positive response; and (3) the animal’s movement needs to be restricted. Currently, two methods are used to restrict animal movement during the test: (1) holding rodents in the hand to control their movement [49,50,51,52,53,54]; and (2) using a customized chamber or other devices to hold the animal but expose its head and face for testing [24,31,55,56,57,58,59,60]. 

Holding rodents in the hand during the von Frey test is not easy. To increase its feasibility, pain researchers at the University of Maryland Dental School improved this method in rats. They put the rat on a soft mat and touched it with a gloved hand for half an hour so that the animal could adapt to the smell of the glove. Next, the rat was poked with von Frey filaments on different facial skin areas. Three or more head withdrawals in five trials are considered positive [50,51,54]. Although this method is difficult for mouse testing, several groups have used it successfully [52,53]. 

On the other hand, using a customized chamber or other devices to restrict animal movement during the von Frey test is much easier. For example, a plastic chamber about 7 cm in all directions with an opening as wide as 2.5 cm has been used to assess facial allodynia [55]. In brief, mice were acclimated to the chambers for 30 min prior to baseline testing. When the animal put its face out of the opening, the investigator stimulated the face twice on either side alternately. The animal’s response was scored from 0 to 4: 0 (no response), 1 (facing to the stimulus or a slower head turn away from the stimulus), 2 (a rapid withdrawal that may or may not be followed by a single face wiping), 3 (attacking or biting the filament or rapid withdrawal followed by 2 face wiping), and 4 (a rapid withdrawal with multiple face wiping) [55]. Another study [56] used a similar device, and the mouse was placed in a three-walled box with an opening, and only the head and front paws could be extended out. The investigator started the testing with the lower-force von Frey filament, and the strong and brisk head movement away from the stimulus was considered a positive withdrawal response [56]. 

In our studies [9,24,31,45,46,47,48], we put the mice in a 10 cm-long Plexiglas cylinder restrainer, allowing the animal to extend its head and front paws but not to rotate in the cylinder. After 1 h of acclimatization, the von Frey filament was applied to the facial skin innervated by the trigeminal nerve in an escalating order, and the animal’s sharp head withdrawal was considered a positive response. Each filament was slightly bent to the skin area and applied 5 times at 10 s intervals. The head withdrawal threshold was calculated as the force at which the positive response occurred in 3 or more of 5 stimuli. 

In a previous study [60] using a closed metal box for the von Frey test, mice were placed in a small box (3” × 3” × 4”) with the top, bottom, and four walls made of black wire mesh (see the box information in [60], in which the animals could move freely. The von Frey filaments were applied to the facial skin areas innervated by the trigeminal nerve branches V2 or V3, and a brisk or active withdrawal of the head from the filament was defined as a positive response [60]. In addition, a method using a small elevated platform (see the platform information in [58] has been developed for the von Frey test in rats. The investigator placed the rat on a small elevated platform and stimulated the rat’s periorbital area with von Frey filaments to measure mechanical allodynia [58].

Typically, different approaches, including “up-down”, “ascending stimulus”, and “percent response” methods, have been used in manual von Frey testing [41]. For the “up-down” method, the 50% withdrawal threshold was determined using Dixon’s method [61]. For the “ascending stimulus” method, von Frey filaments were sequentially applied to the paw or facial skin area in order of increasing force, and each filament was used 5 times, and the paw or head withdrawal threshold was determined as the force at which the positive withdrawal response was produced in 3 of the 5 stimuli [24,41]. For the “percent response” method, the number of positive responses for each filament was converted into a percentage response [41]. The most widely used von Frey testing method is the “up-down” method as utilized by Dixon in human patients and in the measurement of tactile allodynia in rats [62]. In the latter study, von Frey filaments from 0.41 to 15.1 g were used to stimulate the hindpaw of rats with neuropathic pain, and the percentage of paw withdrawal responses was measured with different filament stimulations. Testing was started with 2.0 g of filament (in the middle of the series of von Frey filaments). If a negative response occurred, a heavier von Frey filament with stronger stimulation was applied; if a positive response occurred, a lighter von Frey filament with weaker stimulation was applied. 

Compared with the manual von Frey test, the electronic von Frey test uses only one unbending fiber that has the ability to apply different forces on the animal’s skin. The force that induces withdrawal behavior as described above is recorded as the mechanical threshold [41]. The electronic von Frey system consists of a stimulator handle with sensors, a control unit, and corresponding software [63]. The testing principle is basically the same for both manual and electronic von Frey methods. Although the electronic von Frey test has been used in animal paw withdrawal measurement [63,64,65,66], so far there is no research that uses it to assess orofacial pain in rodents.

*The advantages and disadvantages of the von Frey test method:* The advantages include: (1) It is sensitive and easy to use; (2) the calculation and measurement criteria are straightforward; and (3) the cost of the test setup is relatively low. The disadvantages include: (1) this method may not test pain with dynamic change; and (2) animal activities must be restricted, which could cause stress to the animal and then affect the test results.

The von Frey test for measuring evoked pain has good predictive validity [67,68], especially when it is used to test paw withdrawal behavior in rodent models.

### 3.2. Operant Behavior Test

It is well known that a plantar thermal test with a radiant heat source can be used to measure paw withdrawal latency. However, this method is not usually used for orofacial pain measurement, although a few studies have reported its feasibility [44,69]. Alternatively, facial thermal pain can be assessed with an operant behavior test system with thermoelectric metal probes. This system has been often used in animal pain research [70,71,72,73]. 

The operant behavior test device consists of a test cage with an opening on one side of the wall and a drinking water bottle placed outside the cage. The opening on a wall is surrounded by a circle of thermoelectric probes. When an animal drinks water, its head needs to pass through a metal ring with thermoelectric metal probes to drink from the water bottle connected to a circuit. The duration and frequency of drinking are dependent on the temperature of the metal probes, which can be recorded and analyzed to reflect the stimulus-response of orofacial pain [71,72,73]. Pain testing with the operant system is to determine pain-related aversion and reward activities [70]. The temperature of the thermometer in the operant device can be quickly changed, so the difference in the animal’s response to temperature precisely reflects the level of its facial pain [70,74]. To get stable testing results, animals need to be fasted overnight before testing and also need to receive enough training to allow them to be familiar with the process of pressing their heads through heated metal rings for a liquid reward. 

*The advantages and disadvantages of the operant behavior test method:* The advantages include: (1) The operant system can be connected to multiple cages, thus allowing it to measure facial thermal pain in a large number of animals at the same time; (2) the system can be used to test facial mechanical pain as well; (3) animals can move freely without stress during the testing; and (4) the system automatically records and analyzes the behavior data, and the results are objective and reliable. The disadvantages include: (1) animals need to be fasted before testing; (2) animal training requires on average two weeks of time (3 times/week) [74]; and (3) animal preferences for different types of liquid affect the test results [74].

Although the machines used in operant behavioral tests are not the same, operant tests have high validity for predicting orofacial pain, which is related to the specific design of operant conditioning [75,76,77]. 

## 4. Behavioral Assessment Using Functional Testing 

### 4.1. Dolognawmeter

The dolognawmeter was originally created by Dr. Schmidt and his colleagues to measure gnawing function in mice with TMJ pain [78]. In our previous study, we used the dolognawmeter to measure functional allodynia in inflammatory TMJ pain following intra-TMJ injection of CFA in mice [9]. The principle of this method is to test orofacial pain by assessing mouse biting function. In brief, a mouse is placed in a confinement tube (180 mm long and 24 mm in diameter) with two consecutive blocking dowels to prevent the mouse from escaping, forcing the animal to completely bite through both of the dowels to escape from the opening of the tube. The two consecutive blocking dowels have different diameters, the first being 9 mm and the second being 7 mm in diameter, to facilitate the animal’s confident escape during training. When an animal bites off any dowel, the digital timer connected to the dowel automatically stops and shows the time spent gnawing through each dowel. The baseline is taken as the average of the last three training sessions of a total of six times of training [9,78]. In addition to models of TMJ arthralgia, the dolognawmeter has also been used to measure pain behavior related to oral cancer in mouse models [79]. Additionally, in mouse models of pulpitis and apical periodontitis, the dolognawmeter has been used to show that overexpression of tumor necrosis factor-alpha (TNFα) causes functional allodynia and pain-induced gnawing dysfunction [80].

*The advantages and disadvantages of the dolognawmeter method:* The advantages include: (1) this method shows oral dysfunction produced by orofacial pain; and (2) the test results are objectively and automatically recorded. The disadvantages include: (1) the training sessions take a long time before testing; and (2) some animals cannot be used in the test even after receiving training and must be discarded.

The dolognawmeter can be used for detecting orofacial pain in animals with oral dysfunction. However, we need to make sure that animals are not showing depression or anxiety during training [78].

### 4.2. Bite Force Test

Bite force was originally designed to examine motor function [81]. Since bite force is related to the occlusal function of animals, it was later used to measure TMJ pain, dental pain, and other types of oral pain [12,81,82,83]. In this test, animals require restraint but are allowed to bite the coated strain gauge, and the bite force and duration are recorded through a transducer that connects to the data acquisition system [81]. The observation indexes include biting time, frequency, and amplitude [83].

In a rat model of masseter myalgia [84], the bite force and biting success rate decreased significantly on days 1, 2, and 3 after CFA injection, and the bite force gradually recovered two weeks after CFA injection. The time course of bite force reduction is basically similar to that of CFA-induced masseter myalgia, thus the bite force test can be used to measure orofacial myalgia [84]. This method can also be applied to the mouse test. In mouse models of TMJ pain, decreased bite force was reliably observed, and the bite force was inversely related to the level of TMJ pain [10,82,85]. Dr. Ren and colleagues modified the bite force device by using a 60 mL plastic syringe as a cylinder restrainer for mice. The tip of the syringe was cut open to allow the mouse to protrude its head out. The plunger of the syringe was put back to prevent the animal from escaping. After habituation with the restrainer, the syringe containing the mouse was held manually and moved slowly toward the biting head to start the test. The bite head is composed of a pair of aluminum plates connected to a force–displacement transducer. The accompanying software analyzes the behavior of voluntary biting [10]. 

*The advantages and disadvantages of the bite force test method:* The advantages include: (1) the test is objective; and (2) the bite force change indicates oral dysfunction caused by orofacial pain. The disadvantages include: (1) the animal’s bite force may not be recorded due to the force detection range of the system; and (2) the bite force reduction may not correlate with pain behaviors in the later phase of chronic pain [10].

Similar to the dolognawmeter, the bite force test can be used for detecting orofacial pain in animals with oral dysfunction. Specifically, the changes in bite force intensity and relevant patterns are suitable for evaluating dental pain syndromes [83,86].

### 4.3. Light Aversion Test

It has been reported that during migraine attacks, up to 90% of patients have photophobia [87]. As photophobia is a common symptom of migraine, it is very important to evaluate light aversion behavior in animal models of migraine. To test whether light aversion is developed in a migraine model, a commercially available place preference apparatus including a light box and a dark box has been used [88,89,90]. While rodents normally have an innate aversion to bright areas [91], the aversion toward light may be enhanced in rodents with migraine. 

In our previous studies [47,48], we prepared a migraine model by intraperitoneally injecting nitroglycerin (NTG) into mice, and a 30 min light aversion test was performed immediately following the injection. A modified device including two chambers—a chamber with LED light and a dark chamber—was used in our studies. Mice have free access to the two chambers. A video camera with software was used to record and calculate the percentage of time spent in the dark chamber. Our results show that the mice with NTG-induced migraine spend significantly more time in the dark chamber, suggesting that the mice display migraine-like light aversion behavior [47,48]. Interestingly, in the rat model of NTG-induced migraine, a single injection of NTG does not produce light aversion behavior, while repeated injections of NTG five times within two weeks significantly increase the time spent in the dark [88]. It is important to note that we tested light aversion behavior in mice immediately following NTG injection, whereas the study with a single NTG injection in rats performed a light aversion test at 1 h after NTG injection.

Moreover, a single injection (i.p. or i.c.v.) of calcitonin gene-related peptide into mice obviously induced light-aversion behavior [90], and an intradermal injection of capsaicin in one whisker pad produced photophobia in obese mice [89]. These studies suggest a strong link between trigeminal nerve activation and light-aversion behavior. It has been reported that bright light can increase trigeminal nerve activity via an intraocular mechanism mediated by a luminance-responsive circuit [92]. Of note, it has been purported that there are sex differences in rodents regarding their aversion to light [93,94]; however, the results have been inconsistent between studies [95]. 

*The advantages and disadvantages of the light aversion test method:* The advantages include: (1) In this test, animals do not need training; (2) the test is objective, and the spontaneous behavior of animals is recorded. The disadvantages include: (1) the test results can be influenced by an animal’s movement ability; and (2) the light aversion behavior may be associated with non-painful conditions, such as anxiety. 

The light aversion test is mainly for detecting migraine headaches because photophobia is a typical symptom in migraineurs. It is not suitable for measuring pain behaviors in other orofacial pain, such as TMJ pain [96,97].

## 5. Conclusions

Behavioral testing for orofacial pain is a challenge to pain researchers. Different methods are available for them to use. Here, we provide our suggestions regarding how to select appropriate behavioral testing methods for different types of orofacial pain (Table 2). For measuring spontaneous or ongoing orofacial pain, the grimace scale, facial grooming, CPP, or RTPP can be selected based on which pain model is used in your study. For measuring evoked pain, the manual or electronic von Frey test and the operant behavior test are commonly used in rodent pain models. In addition, the dolognawmeter, bite force test, and light aversion test can be used to assess functional phenotypes or accompanying symptoms in orofacial pain.

## Figures and Tables

**Table 1:** Timeline of CPP test.

Preconditioning Phase (without Optogenetic Stimulation)			Conditioning Phase (Paring with Optogenetic Stimulation)			Testing Phase (without Optogenetic Stimulation)
Day 1	Day 2	Day 3	Day 4	Day 5	Day 6	Day 7

**Table 2:** Pain assessment methods for different orofacial pain in animal models.

Orofacial Pain	Animal Models	Pain Assessment Methods
Myofascial pain	CFA injection into masseter muscle	Facial grooming, operant behavior test, bite force test
TMJ pain	CFA injection into TMJ	Grimace scale, von Frey test, dolognawmeter
Orofacial neuropathic pain	CCI-ION	Facial grooming, von Frey test, CPP or RTPP
Migraine-like pain	Systemic injection of NTG; Dural injection of inflammatory soup	Grimace scale, von Frey test, light aversion test

## Data Availability

Not applicable.

## Abbreviations

CFA: complete Freund’s adjuvant; CPP, conditioned place preference; MGS, mouse grimace scale; NTG, nitroglycerin; RGS, rat grimace scale; RTPP, real-time place preference; TMD, temporomandibular disorders; TMJ, temporomandibular joint; TNFα, tumor necrosis factor alpha.
